# Plasma steroid-binding proteins: primary gatekeepers of steroid hormone action

**DOI:** 10.1530/JOE-16-0070

**Published:** 2016-07-01

**Authors:** Geoffrey L Hammond

**Affiliations:** Departments of Cellular & Physiological Sciences and Obstetrics & GynaecologyUniversity of British Columbia, Vancouver, British Columbia, Canada

**Keywords:** albumin, sex hormone-binding globulin, corticosteroid-binding globulin, testosterone, estradiol, glucocorticoids, progesterone, serine protease inhibitor

## Abstract

Biologically active steroids are transported in the blood by albumin, sex hormone-binding globulin (SHBG), and corticosteroid-binding globulin (CBG). These plasma proteins also regulate the non-protein-bound or ‘free’ fractions of circulating steroid hormones that are considered to be biologically active; as such, they can be viewed as the ‘primary gatekeepers of steroid action’. Albumin binds steroids with limited specificity and low affinity, but its high concentration in blood buffers major fluctuations in steroid concentrations and their free fractions. By contrast, SHBG and CBG play much more dynamic roles in controlling steroid access to target tissues and cells. They bind steroids with high (~nM) affinity and specificity, with SHBG binding androgens and estrogens and CBG binding glucocorticoids and progesterone. Both are glycoproteins that are structurally unrelated, and they function in different ways that extend beyond their transportation or buffering functions in the blood. Plasma SHBG and CBG production by the liver varies during development and different physiological or pathophysiological conditions, and abnormalities in the plasma levels of SHBG and CBG or their abilities to bind steroids are associated with a variety of pathologies. Understanding how the unique structures of SHBG and CBG determine their specialized functions, how changes in their plasma levels are controlled, and how they function outside the blood circulation provides insight into how they control the freedom of steroids to act in health and disease.

## Introduction

Upon their release from steroidogenic cells, biologically active steroids are transported in the blood largely by albumin, sex hormone-binding globulin (SHBG), and corticosteroid-binding globulin (CBG). Additionally, these proteins regulate the non-protein-bound or ‘free’ fraction of steroid hormones in plasma, and control their ability to leave the blood vessels within tissues and to access their target cells (Siiteri *et al.* 1982).

Albumin binds all classes of steroids with low (µM) affinity, but its very high plasma concentrations and ligand-binding capacity allow it to buffer fluctuations in steroid levels and their distribution between other steroid-binding proteins and the free fraction in plasma. Unlike aldosterone, which is bound primarily by albumin, other steroid hormones bind to CBG and SHBG with high (nM) affinity and specificity, with SHBG binding the major androgens and estrogens, and CBG binding the glucocorticoids and progesterone, preferentially (Westphal 1986). Although CBG and SHBG are present in much lower concentrations in plasma than albumin, their high affinity and specificity for steroids enables them to play much more dynamic roles in determining the plasma concentrations of their main ligands. In addition, they control the amounts of free steroids that passively diffuse into cells, and they accomplish this in distinct and diverse ways (Hammond 2011, Perogamvros *et al.* 2012).

The liver is responsible for plasma SHBG and CBG production, but their genes are also expressed in several other tissues where their protein products function differently than in the blood (Hammond 2002, 2011). Programmed fluctuations in plasma SHBG and CBG levels occur throughout development (Scrocchi et al. 1993a, b, Hammond 2011), and abnormal plasma levels of both proteins have been linked to the risk of diseases and their associated pathologies (Hammond *et al.* 2012, Perogamvros *et al.* 2012). Therefore, understanding how the unique structures of SHBG and CBG determine their specialized functions, how changes in their plasma levels are controlled, and how they function outside the blood circulation is integral to understanding how they function as the ‘primary gatekeepers of steroid action’.

## Free steroids are active steroids

The free hormone hypothesis provides a foundation for understanding how steroids act at the target cell level by postulating that only free steroids that are not bound by proteins passively diffuse through the plasma membranes of cells (Mendel 1989). Steroids that are loosely and non-specifically bound to albumin have also been proposed to be accessible to tissues (Pardridge 1988), but steroids still have to dissociate from albumin before they diffuse into cells and exert their activities. Numerous reports of the facilitated uptake of SHBG-bound steroids have also surfaced (Bordin & Petra 1980, Pardridge 1988, Porto *et al.* 1991, Hammes *et al.* 2005), but have never been substantiated in physiologically relevant contexts. At present, the proposition that only free steroids diffuse into cells therefore still best explains the clinical manifestations of either steroid hormone excess or deficiency, and knowledge of free steroid concentrations in plasma is critical to understanding their biological activities.

## Access of plasma steroids to target tissues and cells

While measurements of free steroid concentrations remain the most robust indicator of the biological activities of plasma steroids (Vermeulen *et al.* 1999), adoption of the free hormone hypothesis as a universal explanation for how steroids access their target cells in different tissues and organ systems is overly simplistic (Mendel 1989). This is because steroid-target cells in multicellular organ systems are often compartmentalized and separated from the blood vasculature. Moreover, tissues and organ systems vary enormously in terms of their vascular permeability and the nature of their blood supply, including blood flow and transit time. Extreme examples include the highly fenestrated aspect of the blood vasculature in the liver, where hepatocytes are essentially bathed in blood, vs cells within the brain and testis that are separated by blood barriers. In addition, sex steroid-sensitive epithelial cells in organs such as the prostate, breast, and endometrium are separated from blood capillaries by complex basement membranes, and are compartmentalized together with other cell types (e.g., stroma and adipocytes), in which steroids may either act directly or are metabolically converted into more active hormones in intracrine or paracrine fashions. Thus, the locations of target cells in relation to the blood supply, the endothelial vascular permeability, the composition of the extravascular fluids and extracellular matrix, as well as the juxtaposition of different cell types within a tissue all dictate the ultimate ability of steroids to access their target cells. This review provides examples of how albumin, CBG, and SHBG function in concert with each other, as well as separately, to control the actions of steroid hormones in both the blood and extravascular tissue compartments.

## Albumin

Albumin is the most abundant protein in the blood and it binds steroids and other small lipophilic molecules non-specifically. Its plasma concentrations are normally relatively constant and approximately 1000 times greater than those of the other major steroid-binding proteins (Dunn *et al.* 1981). However, albumin’s affinity for steroids is 3–4 orders of magnitude lower than those of CBG or SHBG (Dunn *et al.* 1981), the plasma concentrations of which undergo much greater fluctuations than albumin, as will be illustrated below. However, reductions in plasma albumin concentrations, which are often seen in patients with severe malnutrition, cirrhosis, the nephrotic syndrome, and other critical illnesses, have been predicted to alter the plasma distribution of testosterone (Dunn *et al.* 1981). Although the mathematical models used in latter study indicated that changes in plasma albumin levels predict only a small effect on the plasma distribution of cortisol, a recent study in critically ill patients has indicated that this introduces a bias in calculations of plasma free cortisol levels (Molenaar *et al.* 2015). Under most conditions, however, where plasma albumin levels are with normal ranges, its main function is to buffer changes in the plasma distribution of steroids when their concentrations increase transiently, or when the production or function of CBG or SHBG change under different physiological conditions or during disease.

## Corticosteroid-binding globulin

Apart from fish, all other vertebrate classes have a plasma protein that binds glucocorticoids and progesterone with high affinity (Westphal 1986). The primary structure of CBG defines it as a clade A serine proteinase inhibitor (SERPINA) family member (Hammond *et al.* 1987) and examination of recent genome databases (www.ncbi.nlm.nih.gov; www.ensembl.org) reveals that *SERPINA6* is in synteny with several other *SERPINA* genes in all mammals; thus, supporting the notion that CBG arose as a result of gene duplications within this cluster of *SerpinA* genes (Billingsley *et al.* 1993). Unlike other SERPINAs encoded by genes within this syntenic gene cluster, CBG (SERPINA6) is not known to inhibit proteases. However, as described in detail below, the specific cleavage of CBG by proteases within a distinct structural domain serves to promote the targeted delivery of CBG ligands to their sites of action.

**Figure 1:**
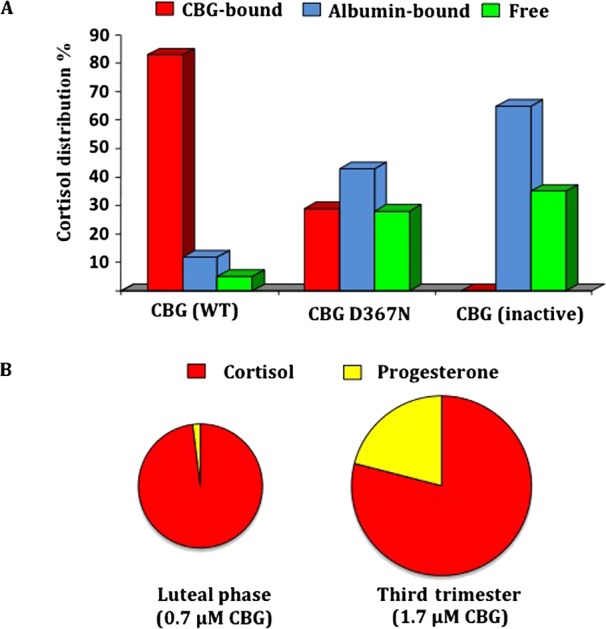
(A) Influence of CBG on the plasma distribution of cortisol. (B) Estimated proportional occupancy of plasma CBG by its major ligands, cortisol and progesterone, in blood samples taken from women before and during pregnancy, and the invervillous compartment of the placenta at term. (A) The plasma distribution of cortisol in individuals with normal CBG (WT); a CBG D367N variant with a four-fold reduction in affinity for cortisol (Emptoz-Bonneton *et al*. 2000), or after heat denaturation to inactivate CBG in a normal human sample (Siiteri *et al*. 1982), were determined by centrifugal ultrafiltration dialysis (Hammond *et al*. 1980). Note that the cortisol distribution in plasma with inactive CBG is expected to resemble that in patients homozygous for naturally occurring CBG variants with undetectable steroid-binding activity, for example, CBG G237V (Perogamvros *et al*. 2010) or CBG W371S (Hill *et al*. 2012). (B) Proportional occupancy of CBG in serum from women during the luteal phase of the menstrual cycle vs the third trimester of pregnancy as estimated computationally from data of serum CBG, cortisol and progesterone levels (Dunn *et al*. 1981).

The most obvious function of CBG in the blood is to transport glucocorticoids (Brien 1981, Perogamvros *et al.* 2012) and recent studies have confirmed that CBG is the primary determinant of circulating plasma cortisol levels in humans (Bolton *et al.* 2014). Perturbations in the plasma distribution of cortisol in patients with CBG variants with reduced affinity for cortisol, for example, CBG D367N (Emptoz-Bonneton *et al.* 2000), or inactive CBG, as observed for CBG G237V (Perogamvros *et al.* 2010) or CBG W371S (Hill *et al.* 2012), illustrate the key role that CBG plays in regulating the free fraction of cortisol in the blood circulation, and how albumin acts to buffer the plasma distribution and free fraction of cortisol in cases of CBG deficiencies (Fig. 1A).

In addition to the liver, *Serpina6* is expressed at relatively high levels in several other tissues, such as the endocrine pancreas and proximal convoluted tubules of the kidney during early development in mice (Scrocchi et al. 1993a, b). These extrahepatic sites of CBG synthesis do not contribute to plasma CBG levels, and the functions of CBG in these locations appear to be distinct from that of plasma CBG. For instance, the CBG produced by the developing rodent kidney is secreted luminally into the proximal convoluted tubules (Scrocchi *et al.* 1993*a*). In this extravascular compartment, it is likely that CBG acts to control the activities of unconjugated corticosterone excreted by the kidneys of immature rodents, at a time when plasma CBG levels are very low due to a delay in their postnatal resumption of hepatic CBG production (Smith & Hammond 1991, Scrocchi *et al.* 1993*a*). Remarkably, although *SERPINA6* (*CBG*) mRNA levels in the developing mouse kidney are greater than in any other tissue, including the liver, at any stage of development (Scrocchi *et al.* 1993*a*), there are no reports of abnormal renal development or function in *Cbg*-deficient mice. Moreover, while some *CBG*-deficient humans present with hypotension (Emptoz-Bonneton *et al.* 2000, Torpy *et al.* 2001), there is also no evidence that they suffer from renal disease.

There is information on the tissue-specific expression of CBG in other animal models during development (Smith & Hammond 1991, Berdusco *et al.* 1995), including baboons (Pepe *et al.* 1996), but changes in *SERPINA6* expression in liver or any other tissues during early human development are unknown. However, as in other mammals, plasma CBG levels are relatively low in human neonates and remain so for the first month (Scott & Wells 1995). The reductions in plasma CBG levels in fetuses and neonates either just before or just after delivery may be important because this will increase the free glucocorticoid levels required for the maturation of organ, such as the lung (Smith & Hammond 1991).

It is widely recognized that CBG regulates the circu­lating levels and plasma distribution of glucocorticoids in mammals. However, during the second and third trimesters of human pregnancy, the large amounts of progesterone produced by the placental trophoblast are capable of displacing glucocorticoids from CBG, and under these circumstances CBG will assume the role of a major plasma progesterone transport protein at least during late gestation (Fig. 1B). In support of this, it has been reported that plasma CBG may influence circulating progesterone levels during human pregnancy and serves as a local regulator of progesterone levels and activity at the maternal–fetal interface (Benassayag *et al.* 2001). This was further illustrated in a study of pregnant Chinese women with a relatively common (frequency of 1:37 in Han Chinese) non-synonymous SNP (rs146744332) that results in the production of a secretion-deficient CBG A51V variant (Lin *et al.* 2012). In the latter study, CBG levels correlated with amounts of circulating progesterone during the first two trimesters of pregnancy, as well as the amounts of progesterone in the intervillous blood (Lei *et al.* 2015). As also reported previously (Benassayag *et al.* 2001), the CBG levels in intervillous blood samples taken from placentas at term vary by almost 10-fold in this study, while the corresponding progesterone levels varied by only three-fold (Lin *et al.* 2012). Moreover, plasma levels of CBG in some intervillous blood samples at term were similar to the corresponding maternal circulating levels, while in others they were as much as four times lower (Lin *et al.* 2012). Importantly, in those intervillous samples with very low CBG levels, the levels of progesterone were about two to three times higher than in the peripheral blood (Lin *et al.* 2012). When this is considered in relation to the estimates of the proportional occupancy of CBG by progesterone in maternal blood in late gestation (Fig. 1B), it can be reasonably predicted that even greater proportions of the CBG in intervillous blood will be occupied by progesterone with more displacement of cortisol, as compared with the maternal circulation, and this will be especially evident in those intervillous samples with low (~100nM) CBG levels.

Although such large differences in intervillous blood levels of CBG must also translate into large differences in the free fractions of both cortisol and progesterone at the maternal interface at term, the physiological significance of this remains obscure, especially because there were no obvious differences in pregnancy outcomes or the health of neonates in CBG-deficient pregnant women as compared with women with normal plasma CBG levels (Lei *et al.* 2015). However, the sex ratio of offspring delivered by women with the secretion-deficient CBG variant (CBG A51V) was significantly female skewed (Lei *et al.* 2015). This latter observation is of interest because stress during pregnancy causes elevations in plasma cortisol, and has been associated with female skewing of the sex ratio at birth (Navara 2010), which may be exacerbated in the offspring of CBG-deficient women. It should also be noted that CBG may act differently in regulating progesterone and or cortisol bioavailability at human fetal–maternal interface in different species, including rodents, where the placenta does not make large amounts of progesterone. In those species, there is a compensatory sustained production of high levels of progesterone by the *corpus luteum* until close to term, and it is possible that CBG plays a role in transporting progesterone of ovarian origin and regulating its actions during early pregnancy, at a time when progesterone has an essential role in supporting blastocyst implantation in all species (Graham & Clarke 1997).

Crystal structure analyses of human and rat CBG showed that the steroid-binding site is located close to the surface of the protein (Klieber *et al.* 2007, Gardill *et al.* 2012, Bolton *et al.* 2014), rather than being buried within its core as previously thought (Defaye *et al.* 1980, Lin *et al.* 2010), and revealed how different steroids interact with specific residues in the steroid-binding site (Lin *et al.* 2010). These experiments also demonstrate how different steroids bind to CBG with high affinity, how proteolysis of its ‘reactive center loop’ (RCL) promotes the irreversible loss of high-affinity binding activity (Lin et al. 2009, 2010), and how naturally occurring mutations of critical residues cause abnormalities in the ability of CBG to bind its ligands (Lin *et al.* 2010, Simard *et al.* 2015). As many as 15 human *SERPINA6* polymorphisms have been characterized with defects in the production or steroid-binding activity of CBG (Simard *et al.* 2015), and these are listed in Table 1. In addition, numerous *SerpinA6* mutations have been reported in animals that were used to study the actions of glucocorticoid in health and disease (Moisan 2010). Knowledge of these CBG variants is important because the algorithms used to calculate free glucocorticoid concentrations in blood samples rely on the assumption that the steroid-binding affinities of CBG in a particular species are constant, and this highlights the need to develop accurate methods to directly determine free plasma glucocorticoid concentrations.

**Table 1:** Non-synonymous *SERPINA6* polymorphisms linked to abnormalities in CBG production or steroid-binding activity.

**SNP** (ID)	**MAF^a^**	**Amino acid^b^**	**Effect on production or steroid binding**	**References**
N/A	N/A	L(5)CfsX26	Translation stop/no production	Torpy *et al.* (2012)
rs777245398	<0.00001	W(11)Stop	Translation stop/no production	Torpy *et al.* (2001)
rs148218218	0.0004	H14R	Produced/Decreased capacity	Simard *et al.* (2015)
rs143058829	0.0002	H14Q	Produced/Decreased affinity	Simard *et al.* (2015)
rs370353870	0.00008	I48N	Low secretion	Simard *et al.* (2015)
rs146744332	0.004	A51V	Low secretion	Lin *et al.* (2012)
rs187253929	0.0004	H89Y	Produced/Decreased affinity	Simard *et al.* (2015)
rs113418909	0.0022	L93H	Produced/Decreased affinity	Smith *et al.* (1992)
rs202107375	0.00007	E102G	Produced/Decreased capacity	Lin *et al.* (2012)
rs754814260	<0.00001	G237V	Produced/No binding activity	Perogamvros *et al.* (2010)
rs201880274	<0.00001	P246L	Produced but not secreted	Simard *et al.* (2015)
rs267604111	N/A	R260L	Produced/No binding activity	Simard *et al.* (2015)
rs374191911	0.00008	1279F	Produced/Decrease affinity	Simard *et al.* 2015
rs28929488	0.0004	D367N	Produced/Decreased affinity	Emptoz-Bonneton *et al.* (2000)
rs267607282	N/A	W371S	Produced/No binding activity	Hill *et al.* (2012)

a Minor allele frequency (MAF) report as the highest frequency among current databases

b Residue numbering is from the amino-terminus of the mature polypeptide sequence (i.e., does not include the 22 residue leader sequence). Amino acids within the leader sequence are indicated in parenthesis.

The observation that C57BL/6J mice are much more sensitive to an acute inflammatory challenge with TNFα when compared with DBA/2J mice enabled the mapping of this response to the murine *Cbg* gene locus in a reference panel of recombinant inbred (BXD) mouse strains derived by crossing C57BL/6J and DBA/2J mice (­Libert *et al.* 1999). This early indication that a pathological response to acute inflammation was linked to CBG made sense because plasma CBG levels in both male and female C57BL/6 had been reported to be about 50% lower than those in DBA/2J mice (Jones *et al.* 1998). The link between low plasma CBG levels and this clinical phenotype was later substantiated in *Cbg (SerpinA6)^–^^/^^–^* mice that were shown to be much more sensitive to an acute ­inflammatory challenge than their wild-type counterparts (Petersen *et al.* 2006). Recently, we have also found that plasma CBG deficiencies in different colonies of Sprague–Dawley rats, which are widely used in studies of glucocorticoid-dependent stress responses and inflammation, are associated with a greater sensitivity to an acute inflammatory challenge (Bodnar *et al.* 2015).

A role for CBG in controlling the activities of glucocorticoids during infectious and inflammatory diseases was suspected from early studies that revealed dynamic reductions in plasma CBG levels in patients with acute infections, traumatic injuries, or severe inflammation (Savu *et al.* 1981, Zouaghi *et al.* 1985, Pugeat *et al.* 1989, Bernier *et al.* 1998), and similar changes were observed in animal studies (Savu *et al.* 1980, Faict *et al.* 1983, ­Garrel *et al.* 1993). The discovery that the gene encoding human CBG is located within a cluster of closely related *SERPINA* genes (Underhill & Hammond 1989, Seralini *et al.* 1990), many of which control proteases or other aspects of inflammatory responses, provided insight into the evolutionary origins of CBG. This also helps to understand why specific proteases target CBG and disrupt its ability to bind steroids, thereby facilitating the release of anti-inflammatory steroids at sites of tissue damage or inflammation (Hammond et al. 1987, 1990, 1991, Hammond 1990).

It is now known that several endogenous proteases, including neutrophil elastase (Hammond *et al.* 1990) and chymotrypsin (Lewis & Elder 2014), and a protease (LasB) produced by the opportunistic bacterial pathogen, *Pseudomonas aeruginosa* (Simard *et al.* 2014), specifically target and cleave the protease-sensitive RCL of CBG disrupting its ability to bind steroids with high affinity. While the significance of RCL cleavage by chymotrypsin is unclear, neutrophil elastase and LasB are present at sites of inflammation and infection, and their ability to specifically target and cleave the RCL of CBG is thought to promote the release of glucocorticoids at these locations.

Plasma CBG levels decrease rapidly in patients and animals undergoing acute inflammation (Savu *et al.* 1981, Garrel *et al.* 1993, Garrel 1996), and it is assumed that this reflects an initial proteolysis of the RCL that will cause a rapid plasma redistribution of glucocorticoids with increases in the albumin-bound and free fractions both locally and systemically. Furthermore, any increases in adrenal glucocorticoid production driven by adrenocorticotrophic hormone (ACTH)-mediated responses to stress can be expected to overwhelm the reduced CBG steroid-binding capacity and accentuate systemic increases in plasma free cortisol levels. It is also known that glucocorticoids (Smith & Hammond 1992) and the pro-­inflammatory cytokine, interleukin (IL)-6 (Bartalena *et al.* 1993), downregulate hepatic CBG production, and increases in their production will act to maintain low plasma CBG levels during the course of inflammation. At some point during the recovery from inflammation, plasma CBG levels are expected to gradually rebound to maintain a normal homeostatic balance of plasma glucocorticoid levels and bioavailability.

Importantly, this model provides a framework for studies of how plasma CBG might be used as a biomarker of the severity of inflammation, and the time course of infectious or acute inflammatory diseases, as well as studies of how pre-existing deficiencies in either the production of CBG or its steroid-binding properties might contribute to poor responses to these diseases. Moreover, given the growing number of human *SERPINA6* polymorphisms that compromise the production or function of CBG (Cizza *et al.* 2011, Simard *et al.* 2015), and the increased frequency of some of these mutations in specific ethnic groups/geographic locations (Cizza *et al.* 2011, Lin *et al.* 2012), the impact of CBG deficiencies on the onset of acute inflammation, as well as the recovery process, needs to be investigated in specific patient groups and animal models.

## Sex hormone-binding globulin

Despite species differences in the way the *SHBG* gene is expressed in the liver during postnatal life, all mammals produce plasma SHBG during critical phases of gonadal and reproductive tract development (Hammond 2011). In human blood, high SHBG levels during childhood likely serve to restrict the premature actions of sex steroids until SHBG declines in both sexes as puberty advances (Hammond 2011, Hammond *et al.* 2012). Serum SHBG levels are generally higher in women than in men, and there is a marked difference in the relative occupancy of SHBG steroid-binding sites between the sexes, with only ~20% of the sites being occupied in women while ~80% of sites are occupied primarily by the much higher plasma testosterone concentrations in men (Dunn *et al.* 1981).

The steroid occupancy of SHBG is further reduced in women taking oral contraceptives that promote large increases in serum SHBG levels, while simultaneously preventing ovarian sex steroid production. It is assumed that the increases in SHBG levels in women taking oral contraceptives reflect increased hepatic SHBG production, because similar five- to ten-fold increases in serum SHBG levels occur in concert with increases in estrogen levels during human pregnancy (Anderson 1974). Increases in serum SHBG in women under these conditions undoubtedly influence the plasma distribution of both androgens and estrogens, and this property has been exploited therapeutically to reduce androgen exposures in women with symptoms of hyperandrogenism (Dewis *et al.* 1985). How estrogens mediate increases in hepatic *SHBG* expression remains to be determined but they most likely function via the estrogen receptorα(ERα (ESR1)) because their abilities to increase SHBG production in HepG2 cells, which are known to be ERα deficient, is increased in a HepG2 cell line (Hep89) engineered to overexpress ERα (Nader *et al*. 2006, Hammond *et al*. 2008). Although the significance of the large increases in maternal plasma SHBG is unclear, transient androgenization has been reported in a pregnant woman with a SHBG deficiency. The fact that androgenic symptoms resolved in this patient, postpartum, suggested that this may be due to an exposure to fetal adrenal androgens that escape placental metabolism, and which would normally be bound by elevated SHBG levels during pregnancy (Hogeveen *et al.* 2002).

Our understanding of the structure and function of SHBG advanced considerably after the crystal structure of the N-terminal laminin G-like domain of SHBG was resolved in complex with a variety of sex steroid ligands (Grishkovskaya et al. 2000, 2002). These high-resolution structures confirmed that androgens and estrogens interact competitively with the same steroid-binding site, but are positioned in opposite and inverted orientations (Grishkovskaya *et al.* 2002), and that each subunit of the SHBG homodimer contained a steroid-binding site (Avvakumov *et al.* 2001). The crystal structures also revealed the location of a calcium-binding site suspected as being essential for both the dimerization and steroid binding of SHBG, and provided insight into how the chelation of calcium in EDTA-treated plasma disrupts these critical structural and functional properties of the molecule (Grishkovskaya *et al.* 2000). In addition, they showed that a zinc atom, positioned at what appears to be an entrance to the steroid-binding site of human SHBG, reduces its affinity for estrogens specifically (Avvakumov *et al.* 2000). This zinc-binding site of plasma SHBG is unlikely to be fully occupied because free zinc concentrations in plasma are very low, but it may be occupied in extravascular tissue compartments, such as the prostate and the male reproductive tract, where zinc levels are exceptionally high.

Crystal structures of human SHBG have also provided unprecedented insight into its ligand-binding site, and how some naturally occurring SHBG variants (Table 2) are produced or function abnormally (Wu & Hammond 2014). Several of these naturally occurring SHBG variants exhibit differences in their relative affinities for either androgens or estrogens, and their positions within the SHBG crystal structure suggest that androgens and estrogens enter or exit the steroid-binding pocket in different ways (Wu & Hammond 2014). The latter observations may explain why androgens and estrogens are oriented so differently within the steroid-binding site (­Grishkovskaya *et al.* 2002).

**Table 2:** Non-synonymous *SHBG* polymorphisms linked to abnormalities in SHBG production or steroid-binding activity.

**SNP** (ID)	**MAF^a^**	**Amino acid^b^**	**Effect on production or steroid binding**	**References**
rs373254168	0.00008	T7N	Produced/Loss of *O*-glycosylation	Wu & Hammond (2014)
rs143521188	<0.00008	T48I	Inefficient dimerization/impaired Ca^2+^ binding/reduced affinity for DHT	Wu & Hammond (2014)
rs373769356	0.00008	R123C	Reduced affinity for DHT/Increased affinity for E2	Wu & Hammond (2014)
rs143269613	0.00008	R123H	Reduced affinity for DHT/increased affinity for E2	Wu & Hammond (2014)
rs368589266	0.00008	R135C	Produced/Increased affinity for E2	Wu & Hammond (2014)
rs6258	0.006	P156L	Produced/Reduced affinity for T	Ohlsson *et al.* (2011)
rs145273466	0.0005	L165M	Produced/Increased affinity for E2	Wu & Hammond (2014)
rs372114420	0.00008	E176K	Produced/Increased affinity for E2	Wu & Hammond (2014)
rs146779355	0.00008	G195E	Low secretion/reduced affinity for DHT	Wu & Hammond (2014)
N/A	N/A	G195R	No secretion	Vos *et al*. (2014)
rs6259	0.09	D327N	Produced/Additional *N*-glycosylation site/normal steroid binding	Power *et al.* (1992)

a MAF report as the highest frequency among current databases

b Residue numbering is from the amino-terminus of the mature polypeptide sequence (i.e., does not include the 29 residue leader sequence).

It has been proposed that SHBG leaves the blood circulation in some tissues and interacts directly with proteins on the plasma membranes of specific cell types, and that this may contribute to either the delivery of SHBG-bound sex steroids via endocytotic mechanisms or to cell membrane receptor-mediated signaling (Rosner *et al.* 2010). We have been unable to confirm these observations, but our studies of mice expressing human *SHBG* transgenes have shown that SHBG does exit the blood vessels in some tissues, and accumulates within extracellular tissue compartments, such as the stroma of the endometrium and epididymis (Ng *et al.* 2006). Moreover, we obtained evidence that this involves a steroid ligand-dependent interaction between SHBG and two members of the fibulin family of extracellular matrix-associated proteins, fibulin-1D and fibulin-2 (Ng *et al.* 2006). The biological significance of this remains to be determined, but it provides *in vivo* evidence that SHBG has the capacity to act in extravascular compartments, extending its functions beyond that of a transport protein that regulates free sex steroids levels in the blood.

Ever since it was realized that the proportions of free testosterone and estradiol in blood samples are inversely related to those of SHBG (Anderson 1974), serum SHBG and testosterone measurements have been used in algorithms to calculate free testosterone levels in patients with suspected hyperandrogenism or hypoandrogenism (Vermeulen *et al.* 1999). Similar relationships between SHBG and free estradiol levels explain how serum SHBG levels contribute to abnormal estrogen exposures in lean women who have high SHBG levels and at risk for osteoporosis (Davidson *et al.* 1982, Devine *et al.* 2005) vs obese postmenopausal women who have low SHBG levels and are at high risk for endometrial cancer (Nisker *et al.* 1980).

The mathematical models used to calculate free plasma androgen or estrogen levels currently rely on SHBG measurements obtained using immunoassays (Vermeulen *et al.* 1999), and they are based on the assumptions that all SHBG molecules react similarly immunologically and have identical steroid-binding properties. These assumptions are fallible, however, because some SHBG variants are not recognized in immunoassays, while others have abnormal affinities for sex steroids (Wu & Hammond 2014), including the SHBG P156L variant with a reduced affinity for testosterone that is present in ~1% of Caucasians, and increases the free fraction of testosterone in the blood of male carriers (Ohlsson *et al.* 2011). These findings highlight the pressing need for sensitive mass spectrometric methods to measure both total and free sex steroid levels in the blood.

This is emerging as an important issue because several other relatively common variations in the human *SHBG* coding sequence are linked to abnormal plasma SHBG levels (Table 2). They include a common non-synonymous SNP (rs6259) that causes a substitution of Asp327 with an Asn residue (D327N) and creates an extra N-linked glycosylation site (Power *et al.* 1992), the utilization of which retards the plasma clearance of SHBG (Cousin *et al.* 1998). Individuals who carry the rs6259 SNP have slightly elevated SHBG levels that have been negatively associated with the risk of developing breast cancer (Forsti *et al.* 2002, Cui *et al.* 2005) or type 2 diabetes (Ding *et al.* 2009). Several polymorphisms in the non-­coding regions of human *SHBG* also appear to influence the hepatic production or blood levels of SHBG (Hogeveen *et al.* 2001, Cousin *et al.* 2004), and numerous reports have associated them with a variety of hormone-related or metabolically related diseases (Xita & Tsatsoulis 2010). There is also a growing awareness that abnormal plasma SHBG levels, and the subsequent changes in the plasma levels and distribution of sex steroids, are not only predictive of numerous clinical conditions, including low bone density (Slemenda *et al.* 1997) and osteoporotic fracture (Cawthon *et al.* 2016) in men, and risk for the metabolic syndrome and its associated diseases in both sexes (Kim & Halter 2014), but may be directly implicated in the etiology of some of these diseases (Legrand *et al.* 2001, Ding *et al.* 2009).

Despite the large number of SHBG measurements performed for diagnostic purposes, it is remarkable that there have been only two reports of complete SHBG deficiencies in humans. An early report of undetectable (<10nM) plasma SHBG levels (Ahrentsen *et al.* 1982) has remained unsubstantiated, but a rare missense genetic mutation that produces a secretion defective *SHBG* variant (SHBG G195R) was recently reported in a young man and his ­sister, both of whom were homozygous for the mutant *SHBG* allele and had no detectable *SHBG* in their blood (Vos *et al.* 2014). As expected, plasma testosterone concentrations in the male proband were well below the normal range, yet his free testosterone levels were normal. Clinical assessments indicated fatigue, overt muscle weakness, and low body weight, and other symptoms of hypoandrogenism, but gonadal development and sperm production and function appeared to be normal. The proband’s affected sibling reported a late menarche and irregular menstrual cycles, but surprisingly had no signs of hirsutism or hyperandrogenism (Vos *et al.* 2014). Although this report provides an indication that plasma SHBG is not essential for male reproductive development and sperm production, the proband’s clinical phenotype suggests a more direct role for SHBG in supporting the anabolic activities of androgens. However, it was noted that this phenotype might be related to possible consanguinity in this pedigree (Vos *et al.* 2014).

Studies of *SHBG* expression in human liver cells in culture (Jänne & Hammond 1998), and in a transgenic mouse model (Jänne *et al.* 1998) indicate that the transcription unit responsible for plasma SHBG production spans a 4.3kb region within the short arm of human chromosome 17 (Berube *et al.* 1990). The sequence flanking exon 1 of this transcription unit lacks a typical TATA box, but the transcription factor hepatocyte nuclear factor 4α(HNF4α) appears to substitute for the TATA-binding protein in helping to recruit the transcriptional machinery to the promoter (Jänne & Hammond 1998). These pioneering studies also demonstrated that a related orphan nuclear hormone receptor (NHR) family member, COUP transcription factor (COUP-TF), competes with HNF4α for the same site in the proximal promoter. While HNF4α actively enhances transcription from this site, COUP-TF represses it, and it is evident that this particular *cis*-­element in the *SHBG* promoter acts together with these two transcription factors as the main on–off switch for *SHBG* transcription (Jänne & Hammond 1998). This provided the first explanation for why low plasma SHBG is a hallmark of the metabolic syndrome and its associated diseases, such as type 2 diabetes and cardiovascular disease (Selva *et al.* 2007). The latter study also challenged the assumption that elevated insulin levels are responsible for the downregulation of *SHBG* expression in hyperinsulinemic/hyperglycemic patients. Instead, this comprehensive study demonstrated that high levels of monosaccharides, especially fructose, repress *SHBG* transcription both *in vivo* and *in vitro* when administered exogenously, and showed that this is mediated by increasing palmitate levels in hepatocytes and a concomitant reduction in HNF4α levels (Selva *et al.* 2007). The mechanism responsible for the loss of HNF4α in hepatocytes under these conditions is unknown, but may somehow be related to the fact that fatty acids, including palmitate (Hertz *et al.* 1998), are ligands of HNF4α (Wisely *et al.* 2002). A direct correlation between hepatic *HNF4-α* and *SHBG* mRNA levels has been also observed in cancer patients, in whom hepatic *HNF4-α* and *SHBG* mRNA levels were reported to be inversely related to hepatic triglyceride levels and to decrease in relation to body mass index (Winters *et al.* 2014).

A variety of hormones and drugs, as well as metabolic and nutritional factors, influence the expression of human *SHBG* and plasma SHBG levels (Table 3). When considered together, the HNF4α emerges as a central mediator of human SHBG production by liver cells. For instance, it mediates the induction of *SHBG* expression in response to thyroid hormone (Selva & Hammond 2009*a*). In this regard, it is interesting that luteinizing hormone (LH) levels rise in hyperthyroxinemic men (Ruder *et al.* 1971) and are high in patients with spontaneous hyperthyroidism (Hudson & Edwards 1992), implying a role for SHBG in the feedback regulation of gonadotrophin-releasing hormone (GNRH) secretion. Elevated plasma levels of pro-inflammatory cytokines (TNF-α and IL-1β) in obese individuals contribute to a reduced hepatic expression of *SHBG* and low plasma SHBG levels by reducing HNF4-α levels in the liver (Simo et al. 2012a, b). Furthermore, adiponectin appears to increase plasma SHBG production indirectly by reducing heptic lipid content and ­increasing HNF4-α levels in the liver (Simo *et al.* 2014). Thus, the low plasma levels of adiponectin that are typically seen in overweight individuals at risk of having the metabolic syndrome (Kadowaki *et al.* 2006) may further contribute to the low plasma SHBG levels in obese patients. By contrast, peroxisome proliferator-activated receptor gamma (PPARγ) appears to interact with a different NHR response element in the *SHBG* proximal promoter to repress transcription in hepatocytes cultured *in vitro* (Selva & Hammond 2009*b*), and this may explain the lower plasma SHBG levels in subjects with a hyperactive 12 Ala PPARγ variant (Mousavinasab *et al.* 2006) that has been linked with low risk for type 2 diabetes and myocardial infarction (Regieli *et al.* 2009). Although this is counterintuitive because the use of potent synthetic PPARγ ligands such as insulin sensitizers, like pioglitazone, cause modest increases in plasma SHBG levels in individuals with the metabolic syndrome (Sridhar *et al.* 2013), the effects of these potent drugs may be multifactorial. Their effects on hepatic SHBG production may be influenced by increases in plasma adiponectin that in turn may increase SHBG production, as previously mentioned. Furthermore, the NHR response element in the *SHBG* proximal promoter that binds PPARγ is also known to bind HNF4-α (Jänne & Hammond 1998), as well as several other orphan members of the NHR family, including constitutive androstane receptor (CAR) and liver X receptor (LXR), which can all potentially compete for binding at this site. This is probably relevant, because pioglitazone and other insulin sensitizers most likely alter the hepatic complement of these orphan NHRs, and these changes may certainly contribute to the modest increases in plasma SHBG levels observed after their administration.

**Table 3:** Hormonal, pharmaceutical, metabolic, and nutritional modifiers of hepatic SHBG production.

**Effector**	**Response**	**Mediator**
Ethinyl estradiol	Increases *SHBG* expression	Mediated by ERα mechanism unknown
	Increases plasma SHBG levels	
Thyroid hormone	Increases *SHBG* expression	Indirect via increased hepatic HNF4-α
	Increases plasma SHBG levels	
Synthetic PPARγ ligands (thiazolidinediones)	Increase *SHBG* expression	Indirect presumably via increased hepatic HNF4-α via reduced hepatic lipids and higher adiponectin levels
	Increase plasma SHBG levels	
Monosaccharides (glucose and fructose)	Decreases *SHBG* expression	Indirect via increased hepatic lipid levels and decreased HNF4-α
	Decrease plasma SHBG levels	
Pro-inflammatory cytokines (TNF-α and IL-1β)	Decreases *SHBG* expression	Indirect via decreased hepatic HNF4-α
	Decrease plasma SHBG levels	
Adiponectin	Increases *SHBG* expression	Indirect via reduced hepatic lipid levels and increased HNF4-α
	Increases plasma SHBG levels	

## Conclusion

Plasma CBG and SHBG are structurally unrelated and function in very different ways that extend well beyond simple transportation or buffering functions in the blood. Their crystal structures have demonstrated how they interact with their preferred steroid ligands, as well as providing insight into other functionally important properties, including the consequences of the RCL cleavage of CBG by specific proteases in the context of infectious and inflammatory diseases, and the impact of dimerization and the binding of divalent cations (Ca^2+^ and Zn^2+^) on the steroid-binding properties of SHBG. Knowledge of the structures together with the identification of naturally occurring variants of CBG and SHBG provide additional insight into their production and functions. They also illustrate the limitations of current methods for measuring their plasma concentrations, which are used in algorithms to calculate free steroid levels, and highlight the need for more direct methods to measure plasma free steroid concentrations. Recent insight into the molecular mechanisms responsible for regulation of hepatic CBG and SHBG production explain how abnormalities in their plasma levels are linked to the risk as well as the consequences of a variety of diseases related to abnormal steroid hormone exposures, and how they may be utilized as biomarkers of disease onset, severity, or recovery. Finally, this review provides several arguments for why CBG and SHBG should be regarded as the primary gatekeepers of steroid hormone action in the blood and extravascular tissue compartments.

## Footnote

This review is based on the 2015 International Medal Lecture, presented by Dr Geoffrey Hammond at the Society for Endocrinology BES 2015, Edinburgh, UK.

## Declaration of interest

The author declares that there is no conflict of interest that could be perceived as prejudicing the impartiality of this review.

## Funding

This study received support from the Canada Research Chairs Program.
